# NADPH–Cytochrome P450 Reductase Mediates the Resistance of *Aphis* (*Toxoptera*) *citricidus* (Kirkaldy) to Abamectin

**DOI:** 10.3389/fphys.2018.00986

**Published:** 2018-08-10

**Authors:** Tian-Xing Jing, Yang Tan, Bi-Yue Ding, Wei Dou, Dan-Dan Wei, Jin-Jun Wang

**Affiliations:** ^1^Key Laboratory of Entomology and Pest Control Engineering, College of Plant Protection, Southwest University, Chongqing, China; ^2^Academy of Agricultural Sciences, Southwest University, Chongqing, China

**Keywords:** NADPH–cytochrome P450 reductase, *Aphis citricidus*, abamectin, heterologous expression, insecticide resistance

## Abstract

NADPH-cytochrome P450 reductase (CPR) plays an essential role in the cytochrome P450 enzyme system, which aids in the metabolism of endogenous and exogenous compounds including the detoxification of insecticides. In this study, the *CPR* transcript in *Aphis* (*Toxoptera*) *citricidus* (Kirkaldy) was cloned, and the deduced amino acid sequence contained an N-terminal membrane anchor, three conserved binding domains (flavin mononucleotide, flavin adeninedinucleotide, and nicotinamide adenine dinucleotide phosphate), a flavin adeninedinucleotide-binding motif, and catalytic residues. Based on phylogenetic analysis, *AcCPR* was grouped in the hemipteran branch. *AcCPR* was ubiquitously expressed at all developmental stages and was most abundant in the adults and least abundant in third instar nymphs. Compared with other tested tissues of adults, the expression level of *AcCPR* was significantly high in the gut. Feeding double-stranded RNA of *AcCPR* reduced the *AcCPR* mRNA level and the activity of AcCPR in aphids, and the treated insects exhibited higher susceptibility to abamectin than the control group. Furthermore, the heterologous overexpression of *AcCPR* in Sf9 cells resulted in a greater viability than control cells when treated with abamectin. All results demonstrated that AcCPR may contribute to the resistance of *A.* *citricidus* to abamectin, and CPR may be a potential target for novel insecticide design or a new factor in the development of insecticide resistance.

## Introduction

The brown citrus aphid, *Aphis* (*Toxoptera*) *citricidus* (Kirkaldy), is an important citrus pest with a global presence and is the most important vector of the *Citrus tristeza virus* (CTV) within its distribution area. The citrus trees can be directly damaged by the sap feeding of *A. citricidus* and the transmission of CTV by the brown citrus aphid can cause the most damage (Hunter et al., 2003; Moreno et al., 2008). Abamectin, a commonly used insecticide, belongs to the avermectin subfamily of macrocyclic lactones (Burg et al., 1979). It is used against major agricultural pests and insects that threaten public health (Lasota and Dybas, 1991; Shoop et al., 1995). It is also widely used to control pest in citrus groves. Therefore, several pest species have developed severe resistance to this insecticide (Liao et al., 2016).

Cytochrome P450 monooxygenase (P450), ubiquitous in all living organisms including insects, is one of the largest super families and plays a key role in the detoxification of xenobiotics such as plant toxins, pesticides, drugs, and mutagens (Feyereisen, 1999). Most biological reactions catalyzed by microsomal P450s requires an integral enzyme, NADPH-cytochrome P450 reductase (CPR) (Paine et al., 2005). In general, each insect species has only one *CPR* transcript, and the CPR protein is thought to be a fusion of two ancestral proteins, a flavin mononucleotide (FMN)-containing flavodoxin and a flavin adeninedinucleotide (FAD)-containing reductase. Therefore, CPR has an N-terminal transmembrane region and the three conserved domains, FMN-, FAD-, and nicotinamide adenine dinucleotide phosphate (NADP)-binding domains (Wang et al., 1997). CPR accepts the hydride ion from NADPH and then transfers electrons to FMN. The step finally reduces the P450 enzyme heme center and activates molecular oxygen (Niu et al., 2017). To date, over 30 CPRs have been identified from insects and most of them are involved in insecticide resistance such as the CPRs from *Bactrocera dorsalis* (Huang et al., 2015)*, Laodelphax striatellus* (Zhang et al., 2016), and *Locusta migratoria* (Zhang et al., 2017).

In the present study, we cloned and characterized the *CPR* gene from *A.* *citricidus*. Expression patterns of *AcCPR* in developmental stages and various tissues were examined by real-time quantitative PCR (RT-qPCR). Using RNA interference (RNAi) to knockdown the expression of *AcCPR*, the susceptibility of *A.* *citricidus* to abamectin was tested. In combination with heterologous expression and cytotoxicity assays in Sf9 cells, the function of *AcCPR* on abamectin tolerance was determined. Our data provided some basic information about CPR in *A.* *citricidus* and elucidated the roles of CPR in the resistance of this aphid to abamectin.

## Materials and Methods

### Insect Culture

The stock colony of *A. citricidus* was originally collected at adult stage from a wild population of aphid in a screen house (Southwest University, Chongqing, China) in 2012. This colony was maintained on potted citrus seedlings (*Citrus sinensis*) in the laboratory at 25 ± 1°C, with 75–80% relative humidity and a 14: 10-h (light: dark) photoperiod.

### Sample Preparation

The aphids used for detecting the relative quantity of *AcCPR* in different developmental stages included the first, second, third (wingless and winged), fourth (wingless and winged) instar nymphs, adults (2-d-old apterous and alate aphids after the final molt), and various adult tissues. The tissues from adult aphids were dissected at 4°C in 0.01 M phosphate buffer saline (PBS). The central nervous system of aphids was dissected from 200 individuals, embryos from 50 individuals, fat bodies from 100 individuals, integuments from 50 individuals, guts from 100 individuals, and salivary glands from 200 individuals, respectively. The adult aphids were used to conduct the RNAi experiment. Four biological replicates were used for all sample preparations. All samples used for RNA extraction were stored in TRIzol reagent (Life Technologies, CA, United States) and frozen at −80°C before use.

### Cloning of *AcCPR*

Total RNA of the collected aphid samples was isolated using TRIzol reagent (Life Technologies) and the first-single-strand cDNA was prepared using the PrimerScript^TM^ RT Reagent Kit (TaKaRa Bio, Dalian, China) according to the manufacturer’s protocol. The open reading frame (ORF) of *AcCPR* was cloned using the primers (Supplementary Table S1) that were designed based on the data of *A. citricidus* transcriptome. PCR was carried out using PrimeSTAR^®^ Max DNA Polymerase (TaKaRa Bio, Dalian, China). The PCR cycling conditions were as follows: 98°C for 3 min; 35 cycles (98°C for 15 s, 58°C for 15 s, 72°C for 2 min); and 72°C for 10 min. The amplified DNA was purified and ligated into the pGEM-T Easy Vector (Promega, Madison, WI, United States). Positive clones were verified by PCR using the universal primers M13F and M13R and sequenced (Invitrogen, Shanghai, China).

### Bioinformatics Analysis of the *AcCPR* Gene

DNAMAN v.6.03 (Lynnon Biosoft, San Ramon, CA, United States) software was used to obtain the deduced amino acid sequence of AcCPR and the ExPASy Proteomics Server^1^ was used to compute the molecular weight and isoelectric point of AcCPR. Conserved domains were predicted using the Conserved Domains Database^2^. The transmembrane helices were predicted using the TMHMM Server v. 2.0^3^. A phylogenetic tree was constructed based on amino acid sequences by the neighbor-joining (NJ) method with 1,000 bootstrap replicates using the MEGA 6.0 (Tamura et al., 2013).

### Quantitative Real-Time PCR (RT-qPCR)

Specific primers (Supplementary Table S1) designed on PRIMER 3.0^4^ were used in RT-qPCR, and the parameters of these primers (efficiency and determination coefficient) were established and calculated. The Bio-Rad CFX Connect^TM^ real-time PCR system was used to perform the RT-qPCR, and this 20 μL-volume system contains 2 × SYBR Green Real-time PCR Master Mix (10 μL), template (1 μL), each RT-qPCR primer (1 μL), and DNase-free water (7 μL). The reaction included an initial 120 s-incubation at 98°C, then 40 cycles (95°C for 30 s, 60°C for 30 s), a step of 60°C for 30 s, and the final step of 95°C for 30 s. Two reference genes, elongation factor-1alpha, and beta actin were used to normalize the levels of *AcCPR* by qBase (Hellemans et al., 2007; Shang et al., 2015).

### Silencing of *AcCPR* by RNAi

The dsRNA primers (Supplementary Table S1) were used to perform PCR reaction to obtain the DNA templates for dsRNA synthesis. The purified DNA templates were then used to synthesize dsRNA according to the TranscriptAid T7 High Yield Transcription Kit (Thermo Scientific, Wilmington, DE, United States).

The *AcCPR* transcript in aphids was knocked down by feeding *dsCPR* through citrus leaves as the description of previous studies in our laboratory (Ding et al., 2017; Shang et al., 2017). Briefly, a citrus stem apex (about 8-cm long) was inserted into a *dsCPR-*containing PCR tube. Then, 25 adult aphids were released onto the citrus stem apex. This *dsCPR-*containing PCR tube with released aphids was moved into a 50-mL plastic tube. The expression level of *AcCPR* was detected after a 24-h dsRNA treatment. Aphids on citrus leaves containing *dsGFP* or PBS were used as controls.

### Bioassays With Abamectin After RNAi

To determine the susceptibility of aphids to abamectin after gene silencing, abamectin was dissolved in acetone (2.5 mg/L, corresponding to LC_50_ of abamectin against brown citrus aphid) and 20 aphids subjected to a 24 h dsRNA treatment were immersed in the abamectin solution for 5 s. After exposure, all the treated aphids were transferred to fresh citrus leaves and maintained under normal rearing conditions. Mortality was determined at 12 h after abamectin exposure. The aphids showing no response or with irregularly shuddering legs were considered as dead. The experiments were repeated four times.

### Heterologous Expression of AcCPR in sf9 Cells

The AcCPR protein was obtained using the Bac-to-Bac^®^ baculovirus expression system (Invitrogen). Briefly, the *AcCPR* ORF was subcloned into the pFastBac HT A vector at the *BamHI/XbaI* cloning sites. The *AcCPR*-HTA bacmid was transformed into DH10Bac competent *Escherichia coli* to obtain the recombinant expression bacmid. Then Sf9 cells (Life Technologies) were transfected by recombinant expression bacmid DNA to produce baculovirus stock. A total of 25 mL Sf9 cells were infected with baculovirus, harvested after infection (72 h) by centrifugation (2,000 × *g*, 10 min), and resuspended with PBS (100 mM, pH = 7.8). The suspensions were sonicated on ice and then centrifuged (15,000 × *g*, 10 min). The supernatant was collected as the recombinant protein sample to measure the activity of CPR enzyme. The control eGFP protein was also expressed using the same method.

### CPR Activity and Cytotoxicity Assays

Heterologously expressed AcCPR activity was determined by spectrophotometric measurement of the activity of NADPH-cytochrome-c reduction according to the previous studies (Guengerich et al., 2009; Huang et al., 2015). Briefly, 166 μL potassium phosphate buffer (0.3 M, pH = 7.7), 16 μL protein sample, 16 μL 0.5 mM cytochrome-c solution (dliluted in potassium phosphate buffer, 10 mM, pH = 7.7), and 2 μL NADPH (10 mM) were mixed thoroughly. Then, we used the Eon Microplate Spectrophotometer (BioTek, Winooski, VT, United States) to record the absorbance (450,600 nm, the slit width of the spectrophotometer was 1.0 nm).

After a 24 h dsRNA treatment, 15 aphids were collected and homogenized on ice with 150 μL potassium phosphate buffer (0.3 M, pH = 7.7). Then the homogenates were centrifuged at 10,000 *g* for 10 min at 4°C and the supernatants were used to test CPR activity using the same method as described above, and the absorbance at 550 nm was recorded for 5 min. The amount (nmol) of cytochrome c reduced per min at 27°C was defined as one unit (Guengerich et al., 2009). The protein content of the supernatants was measured by the Bradford method using a commercial kit (Beyotime Biotechnology, Shanghai, China).

The cytotoxic effect of abamectin on Sf9 cells was evaluated based on a previous study (Wang et al., 2015). For this test assay, 1 × 10^5^ (about 500 μL) infected cells were incubated at 27°C for 24 h in a 24 well plate. Then, 10 μL abamectin at different concentrations (0.625, 1.25, 2.5, 5, 10 μg/mL) was added into each well followed by a 24 h incubation. After incubation, the medium in wells was replaced with 220 μL 3-(4,5-dimethyl-2-yl)-2,5-diphenyltetrazolium bromide (MTT) (20 μL MTT solution mixed with 200 μL fresh medium). After a 4 h incubation, the medium with MTT was replaced with 300 μL dimethyl sulfoxide. Finally, we measured the absorbance of dimethyl sulfoxide solution at 490 nm with the spectrophotometer. The percentage of viable cells relative to cells treated with acetone was calculated as cell viability. In this section, each treatment had four replicates.

### Western Blots

The 12% TGX Stain-Free polyacrylamide gels (Bio-Rad) were used to separate the macromolecules of each protein sample (AcCPR and eGFP). Then Trans-Blot^®^ Turbo^TM^ RTA Mini PVDF Transfer Kit (Bio-Rad) was used to transfer the separated proteins onto a PVDF membrane. After blocking, an overnight-incubation in His tag primary antibodies (Beyotime Biotechnology) at 4°C was followed. Then, the His tag primary antibodies were removed, and the horseradish peroxidase (HRP)-conjugated secondary antibodies (Beyotime Biotechnology) were used to incubate the membrane. Finally, the blotted membrane was imaged after an incubation with Clarity Western ECL Substrate (Bio-Rad) using the ChemiDoc XRS+system (Bio-Rad).

### Statistical Analysis

One-way analysis of variance (ANOVA) was used to test the differences in expression levels of *AcCPR* among developmental stages and among various tissues, and the significance for the means were separated by Tukey’s test (*P* < 0.05). The significance of the difference between two groups was determined by independent samples *t*-tests (*P* < 0.05). All the statistical analyses were performed with SPSS 20.0 for Windows (IBM, Chicago, IL, United States).

## Results

### Cloning and Sequence Analysis of *AcCPR*

**FIGURE 1:**
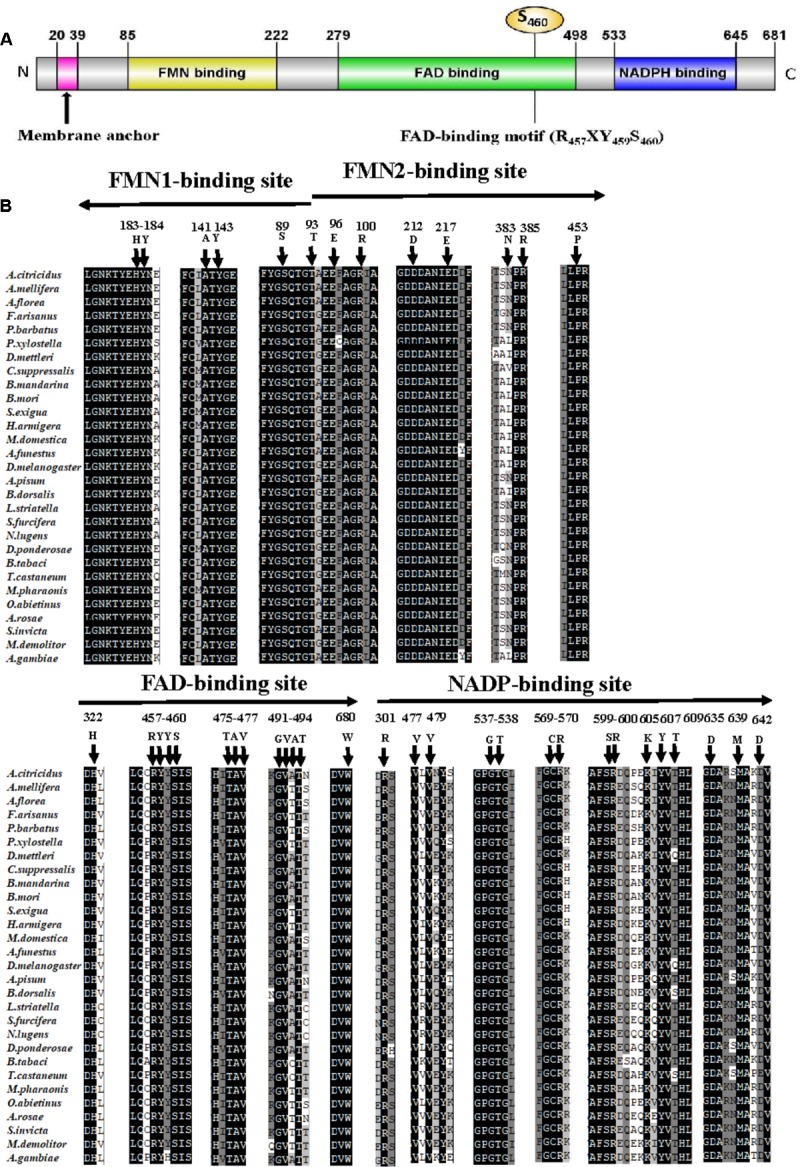
Sequence analysis of the AcCPR protein. Schematic presentation of AcCPR. **(A)** Membrane anchor, FMN-, FAD-, and NADP-binding domains and the FAD-binding motif (R457, Y459, and S460) were showed. **(B)** Alignment of FMN-, FAD-, and NADP-binding sites in insect CPRs. Arrows show the direction from the N-terminus to the C-terminus. All CPR amino acid sequences were downloaded from GenBank (the accession numbers and species names are listed in Supplementary Table S2).

The full-length ORF of *AcCPR* was obtained from the transcriptome of *A. citricidus*. The AcCPR protein with 681 amino acid residues was encoded by 2,046 bp cDNA (GenBank accession no. MG807883). The predicted isoelectric point of this deduced protein was 5.33 with a predicted molecular weight of 77.7 kDa. There was no signal peptide, but a membrane anchor was found in the N-terminal region of AcCPR. It contained a hydrophobic transmembrane motif (20I-39Y: ISALDIALFIVIITVAYFWY), which helps to locate AcCPR on the endoplasmic reticulum. An alignment of AcCPR amino acid sequence revealed that it shared high amino acid identities with CPRs in other insects, including *Acyrthosiphon pisum* (96.6%), *Drosophila melanogaster* (62.6%), *Tribolium castaneum* (62.3%), *Apis mellifera* (60.8%), and *Plutella xylostella* (59.0%). Furthermore, the AcCPR protein possessed several characteristic structural features (**Figure 1**). The key conserved domains of CPR proteins existed across different insect species, including the FMN-, FAD- (three residues R457, Y459, and S456 in this domain), and NADP-binding domains. In addition, four catalytic residues (S460, C633, D678, and W680) in the conserved active sites were also verified.

### Phylogenetic Analysis With Insect CPRs

**FIGURE 2:**
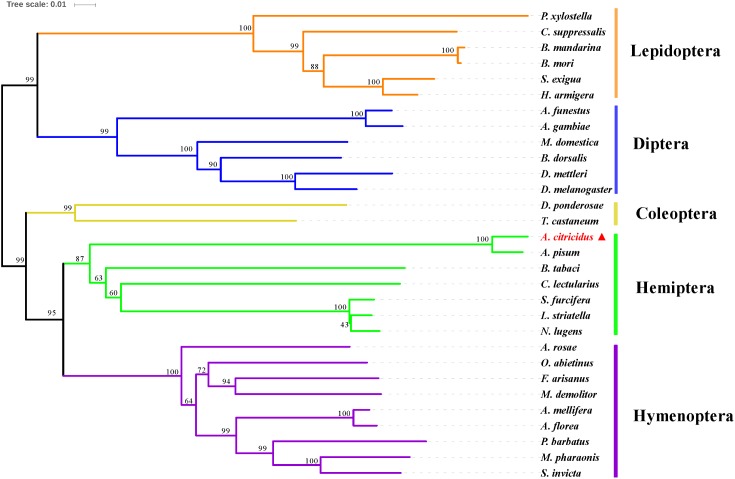
Phylogenic analysis of insect CPRs. AcCPR was marked by filled red triangle. The information about the amino acid sequences used in this phylogenic analysis were given in Supplementary Table S2.

A phylogenetic analysis of AcCPR with 29 other CPR sequences from dipteran, coleopteran, lepidopteran, hymenopteran, and hemipteran insects was performed using MEGA 6.0 (**Figure 2**). The phylogenetic tree showed that CPRs from different insect species can be clearly separated and the CPRs from the same insect orders were grouped in the same branch. AcCPR was grouped in the branch composed of the CPRs of hemipteran insects, including *A. pisum*, *Nilaparvata lugens*, *Cimex lectularius*, and *Bemisia tabaci*, indicating a close evolutionary relationship between AcCPR and other CPRs in the hemipterans.

### Expression Patterns of *AcCPR*

**FIGURE 3:**
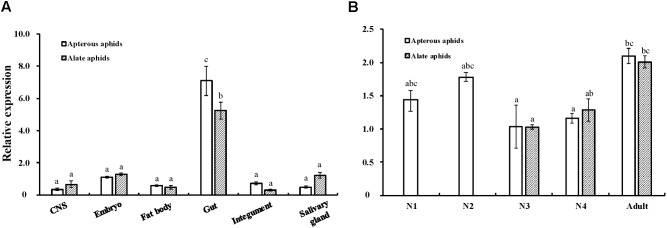
Relative expression levels of *AcCPR* in various tissues **(A)** and at different developmental stages **(B)** of *A. citricidus.* “CNS” refers to the central nervous system; N1–N4, first to fourth instar nymphs; Expression profiles were determined by Quantitative real-time PCR (RT-qPCR). Data were presented as mean ± SE (*n* = 4); different letters denote a significant difference among different samples (*P* < 0.05, one-way ANOVA with Tukey’s test).

The expression patterns of *AcCPR* at different developmental stages and in various tissues were determined using RT-qPCR (**Figure 3**). *AcCPR* was expressed at all the tested developmental stages of *A. citricidus*. The lowest *AcCPR* mRNA level was detected in the third instar nymphs, and it increased from third instar nymphs to adults. The adult aphids (both apterous and alate aphids) had the highest expression levels of *AcCPR*, which suggests that *AcCPR* may play an important role in the adult stage. Among the various tested tissues in adults, the highest mRNA expression was found in gut, while lower expression levels were detected in the central nervous system, embryo, fat body, integument, and salivary glands.

### Sensitivity to Abamectin After *AcCPR* Knockdown

**FIGURE 4:**
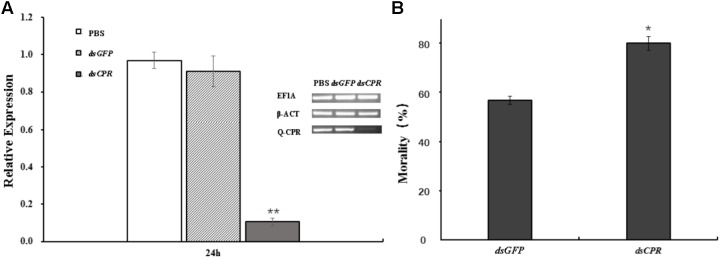
The effect of RNA interference. **(A)** The mRNA levels of *AcCPR* after *dsRNA* feeding was evaluated by RT-qPCR. *A. citricidus* elongation factor-1 alpha (*EF1α*) and beta actin (*β-act*) were used as internal controls. Brightness of electrophoretic bands also showed the expression of *AcCPR* (Q-CPR) and internal control genes (EF1α and β-ACT). **(B)** The resistance of the aphids to abamectin after *AcCPR* silencing. PBS, aphids feeding with PBS; *dsGFP*, aphids feeding with dsRNA against green fluorescent protein; *dsCPR*, aphids feeding with dsRNA against *AcCPR.* The data are the mean ± SE of four biological repeats. One asterisk on the error bar indicates significant differences (*P* < 0.05) and two asterisks indicate extremely significant difference (*P* < 0.01).

The aphids feeding of *dsCPR* specifically decreased the expression of *AcCPR* by 84% compared with the *dsGFP* control (**Figure 4**). Compared with the counterparts in *dsGFP*-treated and PBS-treated aphids, the CPR activity of *dsCPR-*treated *A. citricidus* decreased by 41.8 and 45.4%, respectively (Supplementary Figure S3). Additionally, knockdown of *AcCPR* in the adult *A. citricidus* increased the sensitivity to abamectin exposure significantly. In detail, when treated with 2.5 mg/L abamectin, 78.8% of *AcCPR*-knockdown aphids died and it was a significantly higher mortality than the counterparts of the controls (52.5% for PBS and 54.2% for *dsGFP*, **Figure 4**). Thus, knockdown of *AcCPR* exhibited an impact on the resistance of *A. citricidus* to abamectin.

### Heterologous Expression of AcCPR and Cytotoxicity Assay

**FIGURE 5:**
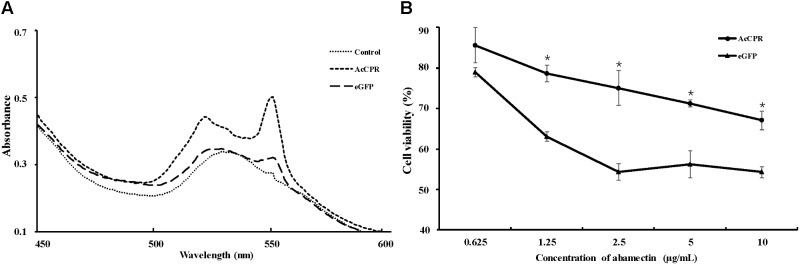
Activity of AcCPR expressed in Sf9 cells measured by cytochrome-c reduction **(A)** and viability of cells to abamectin **(B)**. Control, cell-free extracts of Sf9 cells; eGFP, cell-free extracts of eGFP-expressed Sf9 cells; AcCPR, cell-free extracts of AcCPR-expressed Sf9 cells. One asterisk on the error bar indicates significant differences (*P* < 0.05).

A baculoviral system was used to functionally analyze the AcCPR protein in insect cells. A recombinant *AcCPR* baculovirus stock was produced and transfected into Sf9 cells. Then, the AcCPR fusion protein (6 × His-tagged) was expressed and verified by Western blot (Supplementary Figure S2). The spectral scanning of AcCPR microsomes showed a distinct peak at 550 nm, which indicated that the AcCPR protein heterologously expressed in Sf9 cells had a high activity of cytochrome-c reduction (**Figure 5**).

Based on the successful expression of AcCPR protein with high activity of cytochrome-c reduction *in vitro*, we used the MTT assay to investigate the cytotoxicity effect of abamectin against AcCPR-expressing Sf9 cells. Normalized with acetone-treated cells, the viability of the cells treated by different abamectin concentration was calculated. Compared with the eGFP-expressing Sf9 control cells, the AcCPR-expressed cells exhibited a significantly higher viability than the eGFP-expressed cells at different concentrations of abamectin (**Figure 5**).

## Discussion

Insect P450s play essential roles in insecticide resistance and the detoxification of exogenous compounds. The electron transferring from NADPH to all the microsomal cytochromes P450s was catalyzed by a membrane-bound protein CPR. It has an indispensable role in the CYP/CPR system and is a restricting factor for the P450 activity in the metabolism of both endogenous and exogenous compounds including insecticides. Thus, further studies on the functions of insect CPRs will enrich the understanding of the mechanism responsible for insecticide resistance and promote the process of identification of new insecticide targets. In this study, the full-length ORF of the *CPR* gene from *A. citricidus* was identified (*AcCPR*). The AcCPR protein, with 679 amino acids, shares a high similarity to other CPR proteins, like those of *A. pisum*, *D. melanogaster*, and *Anopheles gambiae.* The phylogenetic analysis showed that *AcCPR* was clustered in the branch containing hemipterans. Structural prediction indicated that the AcCPR protein contains all the conserved CPR structural features, including an N-terminal membrane anchor, FMN-, FAD-, and NADP-binding domains. The hydrophobic N-terminal membrane anchor attaches CPR to the endoplasmic reticulum’s membrane and orients the remaining part of the CPR face on the cytoplasmic side together with the microsomal P450s. This is an indispensable structure for CPR to catalyze the electron transferring to P450s (Laursen et al., 2011). The N-terminus of the CPR protein is the FMN-binding domain containing two binding sites FMN1 and FMN2, and the FAD domain is connected to the FMN domain through a flexible loop. This flexible loop domain enhances conformational changes in the FMN domain to enable interactions with a variety of P450s (Laursen et al., 2011). Moreover, the conserved catalytic residues (S460, C633, D678, and W680) were found and considered as crucial active sites of the hydride transfer reaction.

P450s catalyze various metabolic reactions during the insect life cycle and in different tissues. The catalytic activities of P450s depend on the electrons transferred by the redox partner of P450s, CPR, from NADPH. Therefore, the *CPR* level can reflect the P450 activity. The *CPR* level in *A. citricidus* was relatively higher in the early nymphal and adult stages but was lower in third and fourth instar nymphs. *AcCPR* was expressed in different levels at different stages, indicating that *AcCPR* might be involved in insect developmental processes by mediating the synthesis of 20-hydroxyecdysone driven by insect Halloween P450 enzymes. This mRNA expression pattern was similar to that of *CPRs* in other hemipteran insects, like *L. striatellus* (*LsCPR*) (Zhang et al., 2016) and *N. lugens* (*NlCPR*) (Liu et al., 2015). Such similar expression patterns in hemipteran insects across developmental stages suggest that CPR may have conserved functions in insect development.

*AcCPR* mRNA was also detected in various tissues. The *AcCPR* mRNA quantity was significantly higher in the gut than in other tissues. Among insects, *CPRs* present various expression patterns across tissues and the tissue distribution of genes can give us some clues about their functions. In *D. melanogaster* and *Mamestra brassicae*, *CPRs* are highly expressed in antennae and enhanced their odorant clearance capabilities in insect (Hovemann and Al, 1997; Maïbèche-Coisne et al., 2005). Similar to our results, the CPR in *Helicoverpa armigera* expressed a high transcript level in the midgut. Both *NlCPR* in *N. lugens* and *LsCPR* in *L. striatellus* were present at higher levels in the abdomen than those in other body parts. In general, the above three *CPRs* were involved in xenobiotic metabolism and the development of insecticide resistance (Liu et al., 2015; Zhang et al., 2016). Overexpression levels of *CPRs* respond significantly to insecticide resistance. For instance, the *CPR* transcripts were significantly overexpressed in a fenpropathrin-resistant strain (3.12-fold) of *Tetranychus cinnabarinus* (Li et al., 2015) and isoprocarb-resistant (3.74-fold) strain of *Rhopalosiphum padi* (Wang et al., 2016), respectively. In our study, we also detected the *AcCPR* mRNA level in a relatively abamectin tolerant population (AY) collected from Anyue County, Sichuan Province, China, and this population had a relative higher CPR expression level than the stock population (2.21-fold, Supplementary Figure S1). Many P450 genes are overexpressed 10-fold higher in insecticide-resistant strains than their counterparts in insecticide-susceptible strains, whereas the degree of *CPR* upregulation was not large (<5-fold). Previous studies have indicated that several P450s can be served by a single CPR, and many hypotheses support this idea; for instance, four P450s form a functional “cluster” or oligomer and then served by a single CPR. Also, increased affinity levels of P450 dimers with CPR facilitated a single CPR to serve more P450s (Peterson et al., 1976; Backes et al., 1998; Davydov, 2011). Thus, just a small quantity of CPR can meet the electron requirements of a mass of P450s. That means a small change in CPR may exhibit great influences on P450s, indicating that CPR is a key limiting factor of P450 enzyme activity. This also explains the smaller change in CPR expression relative to those of P450s in insecticide-resistant strains. The overexpression of P450 genes enhances the detoxification of insecticides and the increased metabolic activity requires an enhanced rate of electron transfer catalyzed by CPRs. Thus, the knockdown of CPRs can result in the increased susceptibility to insecticides. This phenomenon has been found in many insects, including *N. lugens* (Liu et al., 2015)*, L. striatellus* (Zhang et al., 2016), and *B. dorsalis* (Huang et al., 2015), as well in our insect of interest, *A. citricidus*, and most researchers support the point that knocking down of CPR reduced the P450 activity and then increased the resistance to insecticides.

Heterologous expression systems can efficiently investigate specific genes’ functions. In *D. melanogaster*, a heterologously expressed insect-specific P450 CYP4G1 was found to be an oxidative decarbonylase and functions in cuticular hydrocarbon biosynthesis (Yue et al., 2012). This system was also widely used in the research on insecticide resistance mechanisms, including the report in which CYP392A16 in *Tetranychus urticae* catalyzed the hydroxylation of abamectin (Riga et al., 2014). In the present study, we overexpressed AcCPR protein in Sf9 cells using the Bac-to-Bac baculovirus system, in combination with the MTT cytotoxicity assay. We found that the AcCPR-overexpressing cells were significantly more viable when treated with abamectin than the control cells (eGFP-expressing cells). Previous studies reported that insect CPRs were related to resistance of many types of insecticides such as pyrethroids (beta-cypermethrin) (Liu et al., 2015), organophosphates (malathion) (Huang et al., 2015), carbamates (carbaryl) (Zhang et al., 2017), and insect growth regulators (buprofezin) (Zhang et al., 2016). However, the relationship between insect CPR and avermectin subfamily is lacking. As a member of avermectin, abamectin is widely used to control insect and mite pests including *A. citricidus* and *Panonychus citri*, and it was found to interact with specific gamma-aminobutyric acid (GABA)-sensitive chloride channels and glutamate-gated chloride channels in nematodes and arthropods (Schaeffer and Haines, 1989; Lynagh and Lynch, 2012). However, some non-target cells can be affected by abamectin. A novel mode of action of abamectin can explain the change in the insect cells’ viability when treated with abamectin. It was reported that abamectin induced apoptosis to kill the insect Sf9 cells (Huang et al., 2011). When treated with abamectin, cytochrome-c, an important apoptosis-induced death effector also the substrate of CPR enzyme, was released from mitochondria into the cytosol during apoptosis when exposed to abamectin (Huang et al., 2011). When AcCPR was overexpressed in Sf9 cells, the cells showed a higher viability. Therefore, AcCPR was probably involved in the progress of apoptosis. The overexpressed AcCPR may act as competitive inhibitor to slow down the process of cell death. Similar mechanisms have been found in two citrus pest mites, *T. urticae* (Lümmen, 2007) and *P. citri* (Leeuwen et al., 2011). Bifenazate is a neurotoxin that acts on the post-synaptic GABA aminobutyric acid receptor. However, studies on these mites suggested an alternative mode in which bifenazate acts as a cytochrome-b ubiquinol oxidation-pocket inhibitor of mitochondrial complex III, and cytochrome-b ubiquinol oxidation-pocket mutations have been proved to be related with the resistance trait in mites. Similarly, once AcCPR acted as a kind of competitive inhibitor of the apoptosis inducer, it may enhance the tolerance capacity of insect cells to abamectin. Although no data are available to prove whether CPR acts as a high effective inhibitor, it may be a new perspective into the action mode of CPR protein in the development of resistance to insecticides.

## Conclusion

In conclusion, this study provides some basic molecular information of *AcCPR* in *A. citricidus*. RNAi through feeding of dsRNA could efficiently silence the mRNA expression of *AcCPR*, decrease the activity of CPR, and enhance the resistance of *A. citricidus* to abamectin. Additionally, overexpression of AcCPR in Sf9 cells led to a higher cell viability upon abamectin treatment. This work expands our understanding of the important physiological role of insect CPR in xenobiotics detoxification and resistance development. Further research is needed to explore the essential role of the *AcCPR* in CYPs-mediated detoxification of insecticides and to test cell death by apoptosis.

## Ethics Statement

The research project was conducted on invertebrate species that were not subjected to any specific ethical issue or legislation.

## Author Contributions

T-XJ, WD, D-DW, and J-JW designed the research and wrote the paper. T-XJ performed all the experiments with the help of YT, B-YD, WD, and D-DW. J-JW provided the materials. T-XJ and YT analyzed the data.

## Conflict of Interest Statement

The authors declare that the research was conducted in the absence of any commercial or financial relationships that could be construed as a potential conflict of interest.

## Footnotes

1 http://cn.expasy.org/tools/pi_tool.html

2 http://www.ncbi.nlm.nih.gov/cdd

3 http://www.cbs.dtu.dk/services/TMHMM/

4 http://bioinfo.ut.ee/primer3-0.4.0/
