# Supplementary material for: NADPH–Cytochrome P450 Reductase Mediates the Resistance of Aphis (Toxoptera) citricidus (Kirkaldy) to Abamectin
Source: Front Physiol. 2018 Aug 10;9:986. doi: 10.3389/fphys.2018.00986 (PMC6104490; doi:10.3389/fphys.2018.00986)
Supplement: Supplementary file 1 [file Presentation_1.PDF]

Supplementary Material

NADPH–cytochrome P450 reductase mediates the susceptibility of *Aphis* (*Toxoptera*) *citricidus* (Kirkaldy) to abamectin

Tian-Xing Jing[1,2], Yang Tan[1,2], Bi-Yue Ding[1,2]，Wei Dou[1,2]，Dan-Dan Wei[1,2] Jin-Jun Wang[1,2]*

[1]Key Laboratory of Entomology and Pest Control Engineering, College of Plant Protection, Southwest University, Chongqing 400715, China.

[2]Academy of Agricultural Sciences, Southwest University, Chongqing 400715, China.

***Correspondence to:** Dr. Jin-Jun Wang, College of Plant Protection, Southwest University, Chongqing 400716, P. R. China. E-mail: jjwang7008@yahoo.com, wangjinjun@swu.edu.cn; Tel: +86-23-68250255; Fax: +86-23-68251269

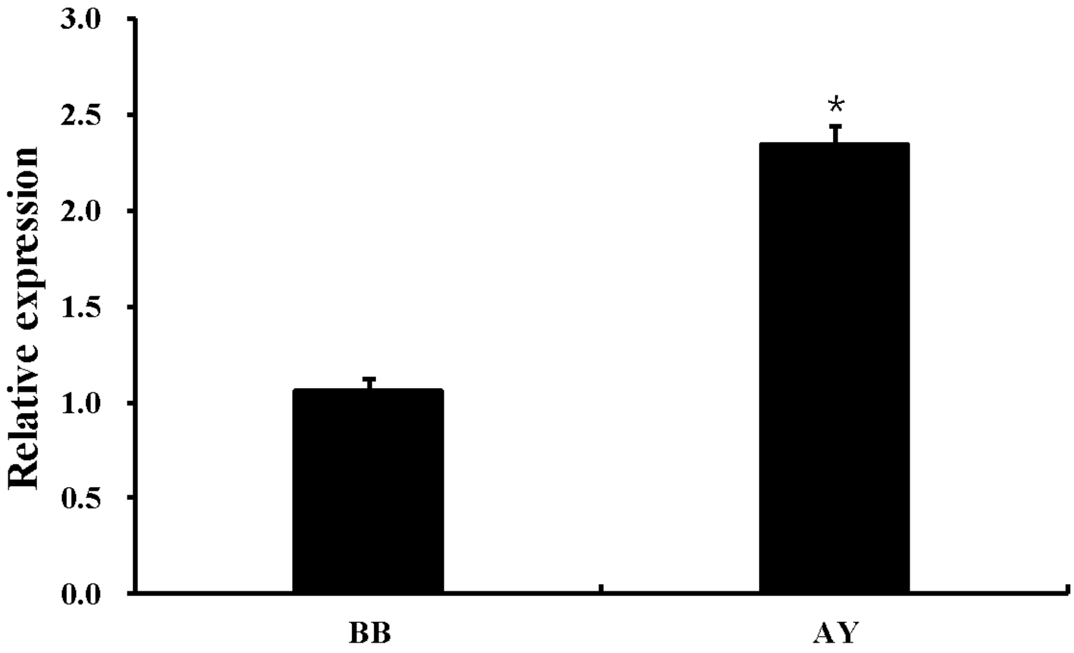

Figure S1. Expression level of *AcCPR* in *A. citricidus* of two different field-populations. The Anyue (AY) *A. citricidus* adults were obtained and collected at an orange grove in Anyue County, Sichuan Province. The LC_{50} ratio (AY:BB) to abamectin is 2.21 fold.

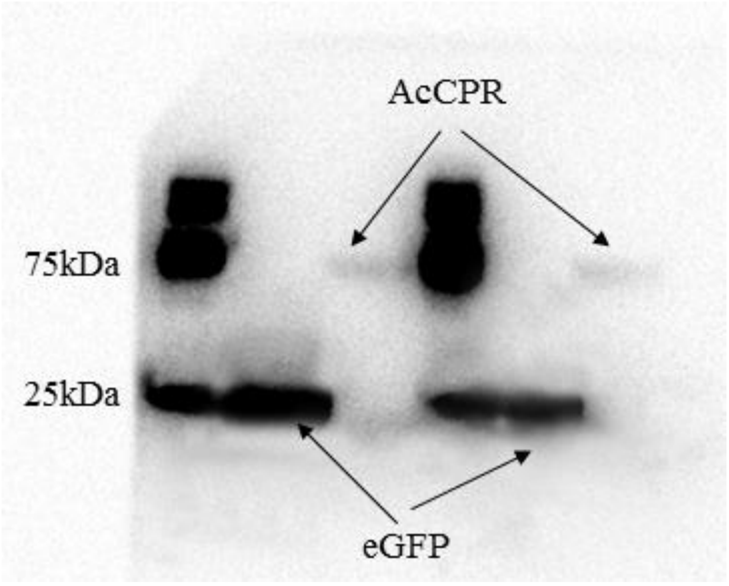

Figure S2. Western blot analysis of eGFP- and AcCPR-expressed Sf9 cells. Two replicates were shown meanwhile.

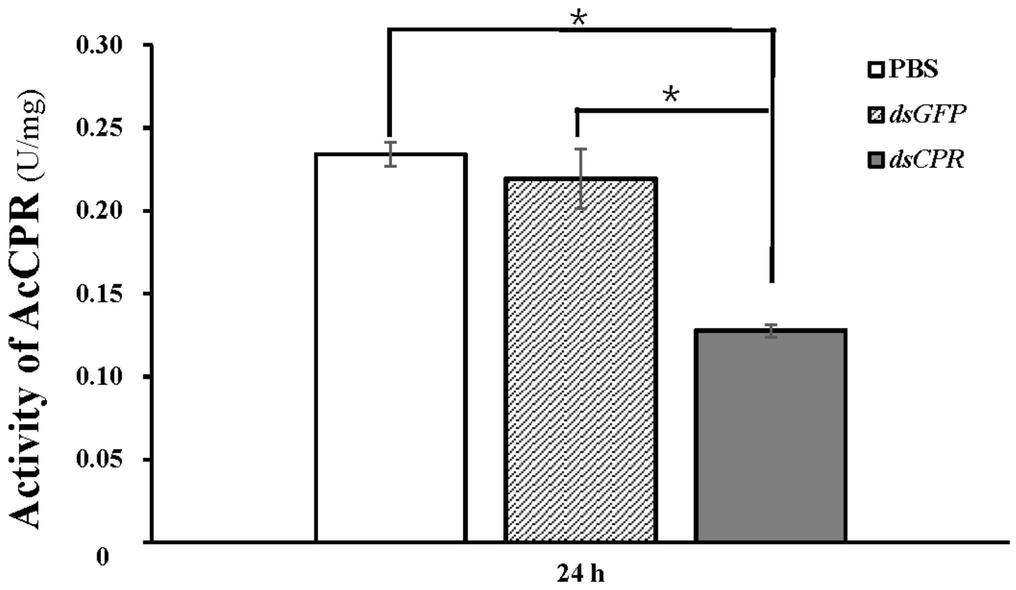

Figure S3. Activity of AcCPR in *A. citricidus* after RNAi. PBS, aphids treated with PBS; *dsGFP*, aphids treated with *dsGFP*; *dsCPR*, aphids treated with *dsCPR.* Asterisk indicates significant differences ($P < 0.05$).

Table S1. Primers used in this study

Application of primers	Primer names	Sequence (5′–3′)	Product size (bp)
ORF cloning	*AcCPR-F*	ggatccgATGGAGAATTCTGAAG	2059
	AcCPR-R	tctagaTCAGCTCCACACATCAGC	
RT-qPCR	*AcCPR-qF*	TTGGGAGATGATGACGCCAA	285
	AcCPR-qR	AGAACGGTCGCCAGATTTGT	
	EF1α-F	GATGCACCTGGTCACAGAGA	194
	EF1α-R	CCATCTTGTTCACACCAACG	
	β-ACT-F	TCCTTCGTCTCGATCTTGCT	214
	β-ACT-R	TGTCCATCAGGCAATTCGTA	
dsRNA synthesis	*dsAcCPR-F*	TAATACGACTCACTATAGGGCGAAGTTTTACCAGAACG	561
	dsAcCPR-R	TAATACGACTCACTATAGGGGGTTATCATGTAAAATCCAATCG	
	dsGFP-F	TAATACGACTCACTATAGGGCAGTTCTTGTTGAATTAGATG	482
	dsGFP-R	TAATACGACTCACTATAGGGTTTGGTTTGTCTCCCATGATG	

Table S2. Insect species of NADPH-cytochrome P450 reductase and GenBank accession numbers

Insects	GenBank accession numbers	Insects	GenBank accession numbers
Acyrthosiphon pisum	XP_001945312.1	*Apis mellifera*	XP_006569767.1
ogatella furcifera	AHM93009.1	*Apis florea*	XP_012341485.1
Nilaparvata lugens	AHB59865.1	*Fopius arisanus*	XP_011306347.1
Dendroctonus ponderosae	AFI45002.1	*Pogonomyrmex barbatus*	XP_011643152.1
Cimex lectularius	AFD50507.1	*Plutella xylostella*	NP_001292469.1
Bemisia tabaci	AGT15701.1	*Drosophila mettleri*	AAB48964.1
Tribolium castaneum	XP_971174.2	*Chilo suppressalis*	AGM20565.1
Monomorium pharaonis	XP_012541364.1	*Bombyx mandarina*	ABJ97709.1
Orussus abietinus	XP_012275162.1	*Bombyx mori*	NP_001104834.1
Athalia rosae	XP_012251354.1	*Spodoptera exigua*	ADX95746.1
Solenopsis invicta	XP_011157063.1	*Helicoverpa armigera*	ADK25060.1
Microplitis demolitor	XP_008548684.1	*Musca domestica*	NP_001273818.1
Anopheles gambiae	AAO24765.1	*Anopheles funestus*	ABO77954.1
Drosophila melanogaster	CAA63639.1	*Laodelphax striatella*	AID55422.1
Bactrocera dorsalis	GU325631	*Aphis citricidus*	MG807883
